# Initiation of Tertiary Lymphoid Structures for Cancer Immunotherapy

**DOI:** 10.34133/research.1164

**Published:** 2026-02-25

**Authors:** Ji-Yuan Zou, Ze-Tong Li, Ze-Cheng Wang, Hao Li, Zhi-Jun Sun

**Affiliations:** State Key Laboratory of Oral & Maxillofacial Reconstruction and Regeneration, Key Laboratory of Oral Biomedicine Ministry of Education, Hubei Key Laboratory of Stomatology, School & Hospital of Stomatology, Frontier Science Center for Immunology and Metabolism, Taikang Center for Life and Medical Sciences, Wuhan University, Wuhan, China.

## Abstract

Tertiary lymphoid structures (TLSs) are ectopic lymphoid aggregates formed by lymphocytes and antigen-presenting cells within chronic inflammatory microenvironments or tumor microenvironments (TMEs), and their initiation phase is a critical step for mounting an effective antitumor immune response. TLS initiation depends on the interplay between lymphoid tissue inducer cells and lymphoid tissue organizer cells and is finely regulated by multiple cytokines and chemokines. This review systematically summarizes the key triggers and signaling networks driving TLS initiation, elucidating how TLSs reshape the TMEs, facilitate antigen presentation, and recruit immune cells to enhance antitumor immune responses. Furthermore, potential strategies to modulate TLS initiation are discussed, along with the emerging role of TLSs as promising targets in cancer immunotherapy.

## Introduction

With the revolutionary breakthroughs of immune checkpoint blockade (ICB) therapy in cancer treatment, immunotherapy has become a central focus of modern oncology [1,2]. Recent research has demonstrated that patient prognosis is closely related to the dynamic characteristics of the tumor microenvironments (TMEs), in which the density and functional status of tumor-infiltrating immune cells have emerged as key predictors of immunotherapy response [3]. Nevertheless, many solid tumors still display an immunologically “cold” phenotype, thereby limiting the efficacy of ICB. Consequently, modulating the TMEs to convert “cold” tumors into “hot” tumors with abundant immune cell infiltration has become a critical objective for improving immunotherapy response rates and enhancing patient outcomes.

Tertiary lymphoid structures (TLSs), as key immune hubs, provide critical support for antitumor responses through antigen presentation and lymphocyte activation [4,5]. Structurally resembling secondary lymphoid organs (SLOs), TLSs are composed of T cells, B cells, dendritic cells (DCs), and high endothelial venules (HEVs) [6]. TLS formation begins with the initiation phase, which is driven by interactions between lymphoid tissue inducer (LTi) cells and lymphoid tissue organizer (LTo) cells through a network of cytokines and chemokines [7]. This initiation phase establishes the framework for lymphocyte infiltration and cytokine signaling, directly influencing the strength and quality of antitumor immunity. Studies have shown that efficient TLS initiation is associated with enhanced responsiveness to ICB and favorable patient outcomes, underscoring its pivotal role in shaping the TMEs and cancer immunotherapy [8].

This review systematically summarizes the initiating signals, dynamic changes, and key roles, while outlining the cellular interactions and signaling pathways involved in the initiation phase. Building on these insights, regulatory strategies for TLS initiation are further discussed. Additionally, the review discusses biomarkers, molecular imaging, and the therapeutic implications of TLS initiation for future clinical applications.

## Initiation of TLSs

TLSs are ectopic lymphoid formations that emerge in non-lymphoid organs under pathological conditions yet lack the encapsulating capsule characteristic of lymph nodes [9]. Unlike SLOs, TLSs do not appear during embryogenesis but form postnatally in response to chronic inflammatory stimuli, and they can arise in diverse settings such as tumors, autoimmune diseases, and other chronic inflammatory environments [10].

The initiation of TLSs begins with immune microenvironment alterations triggered by chronic inflammation or persistent antigenic stimulation [11]. Although there is currently no universally defined boundary for the initiation phase of TLSs, this review considers all events occurring before TLS maturation to belong to the initiation phase. Accumulation of inflammatory factors mobilizes LTi cells, which express lymphotoxin α1β2 (LTα1β2) and tumor necrosis factor (TNF) [12]. These factors bind to lymphotoxin β receptor (LTβR) and TNF receptor (TNFR) on LTo cells, activating canonical and noncanonical NF-κB pathways [13]. This signaling process promotes the secretion of vascular endothelial growth factor (VEGF) A/C) by stromal cells, thereby facilitating the development of HEVs and inducing the expression of various adhesion molecules (VCAM1, ICAM1, and MAdCAM1) [7]. In addition, LTα1β2–LTβR signaling triggers the production of a series of chemokines, primarily CCL19, CCL21, CXCL12, and CXCL13. These chemokines not only induce lymphocytes to express LTα1β2 but also recruit lymphocytes from adjacent HEVs and regulate their entry into the T cell and B cell zones. Additionally, DCs, M1 macrophages, innate lymphoid cells (ILCs), B cells, CD8^+^ T cells, and T helper 17 (Th17) cells can initiate TLS formation in a manner analogous to LTi cells [14,15]. Moreover, cancer-associated fibroblasts, vascular smooth muscle cells, and adipocytes may substitute for LTo cells in certain pathological contexts [16,17].

**Fig. 1.**
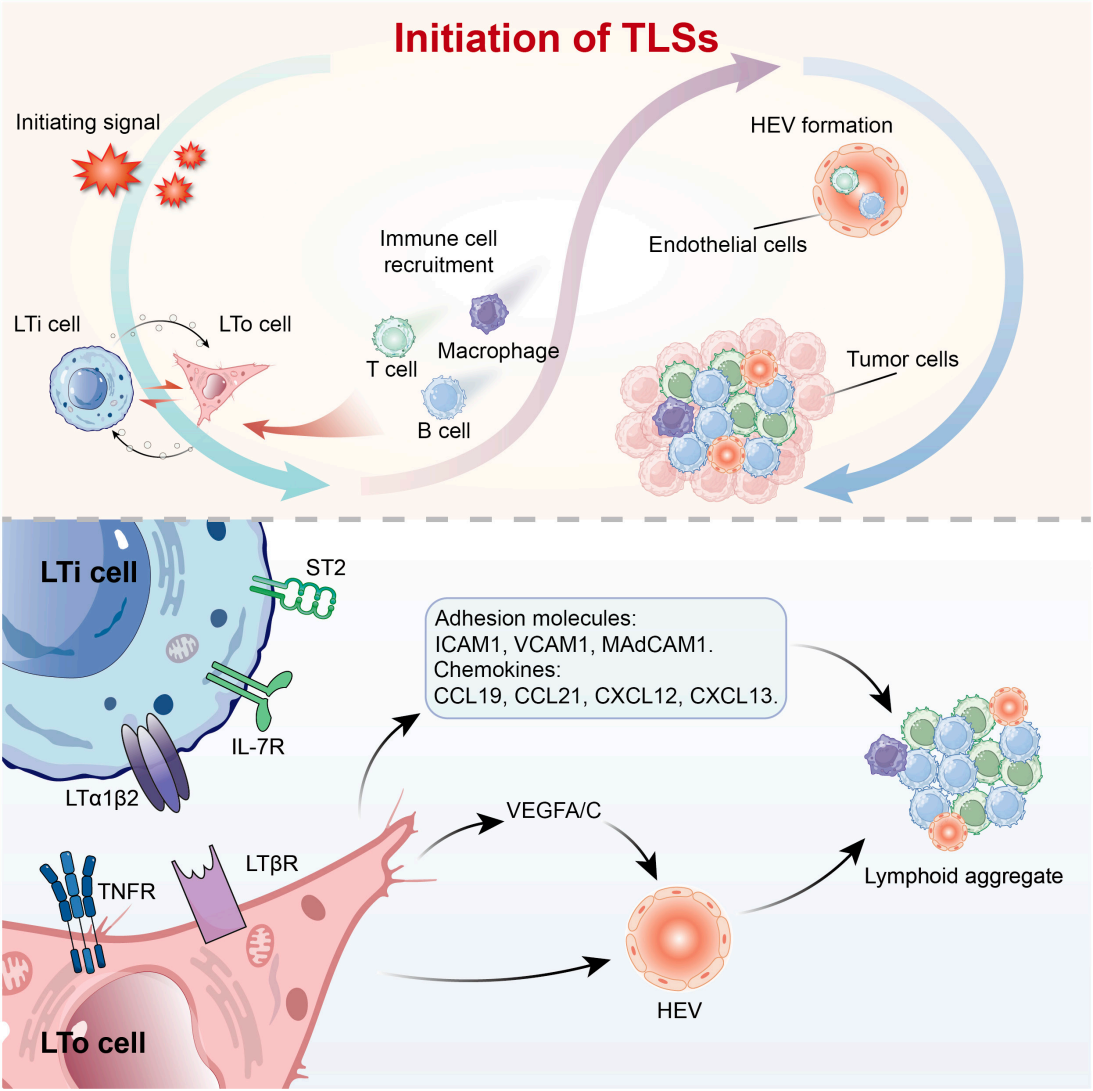
Initiation of TLSs. Initiating signals activate LTi cells, which interact with LTo cells. Activated LTo cells up-regulate adhesion molecules and chemokines to recruit immune cells, while VEGFA/C promotes HEV formation, thereby driving lymphoid aggregation and TLS initiation. HEV, high endothelial venule; LTi, lymphoid tissue inducer; LTo, lymphoid tissue organizer; TLS, tertiary lymphoid structure; VEGF, vascular endothelial growth factor; LTα1β2, lymphotoxin α1β2; LTβR, lymphotoxin β receptor; TNFR, tumor necrosis factor receptor.

The initiation phase of TLSs is mechanistically and functionally distinct from the subsequent maturation stage, as summarized in Table 1. TLS initiation is operationally characterized by the induction of lymphoid chemokines, the appearance of nascent PNAd^+^ HEV-like vessels, early activation of LTβRNF-κB signaling, and the onset of immuneNF-κB signaling, and the onset of immune cell recruitment. In contrast, TLS maturation is defined by clear T and B cell zones, the formation of follicular dendritic cell (FDC) networks, germinal center (GC) reactions, and the generation of memory T and B cells [18,19]. Functionally, initiation establishes a permissive immune microenvironment for lymphocyte entry and priming, whereas maturation confers full cellular and humoral immune competence. Beyond its structural features, the initiation phase of TLSs is a decisive determinant of antitumor immune efficacy. Clinical and transcriptomic studies across multiple solid tumors have demonstrated that tumors exhibiting early TLS features or chemokine niche signatures display enhanced immune infiltration and improved responsiveness to ICB [20,21]. Conversely, impaired lymphocyte recruitment and reduced antigen presentation result in inferior immunotherapeutic efficacy. A schematic overview of the TLS initiation process and its immunological significance is shown in Fig. 1.

**Table 1.** Defining features of TLS initiation versus maturation

Dimension	Initiation	Maturation	Reference
Histologic features	Diffuse immune cell aggregates; nascent PNAd^+^ HEVs; lack of GC or FDC network.	Well-organized T and B cell zones; mature PNAd^+^/CD31^+^ HEVs; presence of CD21^+^/CD35^+^ FDC networks; GC architecture with emergence of plasmablasts/plasma cells (CD138^+^).	[7]
Molecular signatures	High expression of CCL19, CCL21, CXCL12, and CXCL13; activation of LTβR–NF-κB signaling; expression of adhesion molecules (ICAM1, VCAM1, and MAdCAM1).	Full GC signature (AID, BCL6, CD40L, and IL-21 axis); LTβR signaling sustained and structurally stabilized.	[18,19]
Functional characteristics	Early cellular recruitment; limited antigen presentation; priming but not full humoral coordination.	Active GC reactions; generation of memory T and B cells; coordinated cellular and humoral antitumor immunity.	[20]

HEV, high endothelial venule; GC, germinal center; FDC, follicular dendritic cell; LTβR, lymphotoxin β receptor

## Initiating Signals

**Fig. 2.**
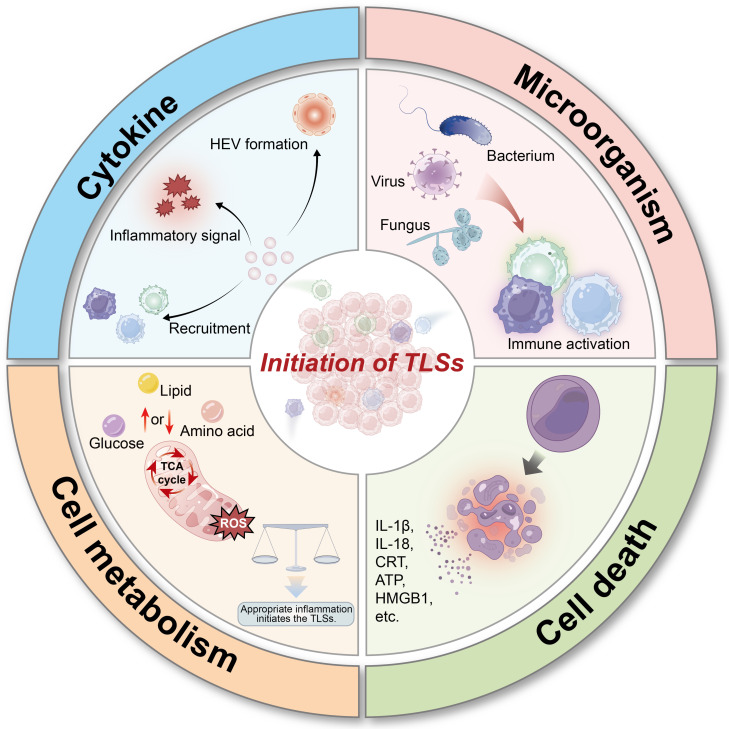
Initiating signals of TLSs. The initiating signals of TLSs include cytokines, microorganisms, cell death, and metabolism. They regulate the initiation of TLS through antigen presentation or the secretion of related cytokines. TLS, tertiary lymphoid structure; HEV, high endothelial venule; ROS, reactive oxygen species; TCA, tricarboxylic acid; ATP, adenosine triphosphate; HMGB1, high-mobility group box 1.

The initiation of TLSs is an adaptive response of the immune system that develops in chronic inflammatory microenvironments or TMEs, and its initiation mechanism involves a multilayered regulatory network, which is regulated by multiple factors [22]. Therefore, understanding the causes of TLS initiation is crucial for immunotherapy. This section systematically summarizes the factors that initiate TLSs and their underlying mechanisms (Fig. 2).

### Cytokine

Cytokines are small molecule proteins that are secreted by immune or non-immune cells and can participate in the immune response [23]. The initiation process of TLSs involves the synergistic action of multiple cytokines and chemokines, which establish the initial microenvironment for immune cell aggregation. LTα1β2 and TNF, which are secreted by LTi cells, bind to LTβR and TNFR on LTo cells, respectively. Together, they promote the secretion of chemokines and adhesion molecules by LTo cells, including CCL19, CCL21, CXCL12, CXCL13, VCAM1, ICAM1, and MAdCAM1. Meanwhile, interleukin-17 (IL-17), secreted by LTi cells, binds to IL-17R on LTo cells, synergizing with the LTα1β2–LTβR signaling axis to drive LTo cells to release chemokines, thereby directing immune cells to aggregate to inflammatory sites. Notably, IL-7 and receptor activator of nuclear factor κB ligand (RANKL), secreted by LTo cells, can in turn bind to IL-7R and receptor activator of nuclear factor κB (RANK) on LTi cells, enhancing the LTα1β2–LTβR signaling axis and forming a positive feedback loop [24]. The signaling axis also promotes the secretion of VEGFA/C by LTo cells, thereby promoting HEV generation.

In addition to the canonical pathway, many other mechanisms can initiate TLSs [25]. For instance, in Sjögren’s syndrome, immunofibroblasts regulate TLS initiation via the ICOS–LTα–CCL19 signaling axis [22]. TNFSF14, the 14th member of the TNF superfamily, is another LTβR ligand, also known as LIGHT [26]. Studies show that the LIGHT–LTβR pathway promotes LTo cell production of CCL21 and activates HEV formation, participating in the initiation phase of TLSs [25]. Furthermore, type I interferon (IFN-I) and IL-1 have been shown to stimulate LTo cells to secrete CXCL13 and recruit B cells to initiate TLSs [27]. A recent study further indicates that IL-33 can induce ILCs to produce lymphotoxin, thereby triggering TLS initiation through activation of lymphoid stromal programs [15]. IL-13 and IL-22, which are mainly secreted by T cells, ILCs, and natural killer (NK) cells, up-regulate the expression of adhesion molecules (ICAM1 and VCAM1) in LTo cells and are key factors during the initiation phase [16].

### Microorganism factors

The activities of microorganisms are closely related to the initiation of TLSs [28]. The chronic inflammatory response induced by microorganisms can remodel the local immune microenvironment, creating favorable conditions for TLS initiation.

#### Bacterium

Inflammation plays a critical role in the initiation of TLSs, and bacteria can modulate the onset and progression of inflammation, which may in turn influence TLS initiation [29]. Pathogen-associated molecular patterns (PAMPs) released by bacteria are recognized by pattern recognition receptors (PRRs) on host immune cells, including Toll-like receptors (TLRs), nucleotide-binding oligomerization domain-like receptors, and retinoic acid-inducible gene I-like receptors [30]. Such PAMP–PRR interactions rapidly activate immune and stromal cells, leading to the release of large amounts of pro-inflammatory cytokines and chemokines. For example, lipopolysaccharide (LPS) from *Fusobacterium nucleatum* can be recognized by TLR4 on immune cells, activating the TLR4/MyD88 cascade and stimulating the NF-κB pathway, thereby inducing inflammation [28]. Si et al. [31] demonstrated that oral administration of live *Lactobacillus rhamnosus GG* (*LGG*) enhanced immunotherapy efficacy by increasing tumor infiltration of DCs and T cells. *LGG* also activated the cyclic GMP–AMP synthase (cGAS)–stimulator of interferon genes (STING) pathway, which may further contribute to TLS initiation [31]. Increasing evidence has also shown that the gut microbiota can promote TLS formation by regulating host immune functions and TLS-associated chemokines [29]. Taken together, these findings indicate that bacteria play a critical role in the initiation of TLSs.

#### Virus

As exogenous pathogens, viruses can initiate TLSs by activating the host innate immune response [32]. Viral nucleic acids and proteins can be recognized by DCs, macrophages, and fibroblasts through receptors such as TLRs, thereby inducing IFN-I and various pro-inflammatory cytokines. Oncolytic viruses (OVs), as a novel class of immunotherapeutic agents, not only release tumor-associated antigens but also recruit T cells, enabling them to specifically target and eliminate cancer cells [33]. Moreover, OVs can activate DCs through the STING pathway, thereby promoting TLS formation [34]. Oncolytic herpes simplex viruses (oHSVs), a subtype of OVs, have been shown to mediate TLS initiation by up-regulating CXCL10/CXCR3 signaling [35]. Dhital et al.[36] further demonstrated that the combination of the JAK protein kinase inhibitor ruxolitinib and oHSV enhanced immune cell infiltration into the TME, changes suggestive of TLS induction. However, contradictory reports exist, showing no association between viral infection and TLS formation, suggesting that such outcomes may depend on viral type as well as the specific tissue microenvironment [37].

#### Fungus

Although there is currently no direct evidence demonstrating that fungi regulate TLS initiation, insights from the molecular mechanisms of fungal infection suggest a potential association [38]. Studies have shown that *Candida albicans* can activate the IFN-I pathway in epithelial cells through β-glucan in its cell wall, and IFN-I in turn induces LTo cells to produce initiation-associated chemokines [39]. Compared with bacteria, fungal cell wall components such as β-glucan and mannans can activate macrophages and DCs through receptors including Dectin-1 and TLR2/TLR4, leading to the secretion of pro-inflammatory cytokines such as IL-1β and TNF-α, thereby initiating immune responses. Under fungal stimulation, fibroblasts can differentiate into CCL21^+^ subsets, which recruit immune cells via secretion of factors such as CXCL12.

These findings indicate that microorganisms participate in TLS initiation and functional regulation through multiple molecular mechanisms. Notably, microbes exhibit a dual role in TLS induction: acting as hubs for antitumor immunity on the one hand, while serving as pathogenic mediators in autoimmune diseases on the other.

### Metabolic factors

Metabolism, as a central regulatory network for cellular function, has a profound impact on the initiation of TLSs through multiple pathways including glucose metabolism, lipid metabolism, and amino acid metabolism [40].

#### Glucose metabolism

Glucose metabolism is closely linked to immune cell function, serving as both a central source of energy and a regulator of signaling pathways, and may contribute to TLS initiation through multiple mechanisms [41]. On the one hand, glycolysis enhances effector T cell activation and pro-inflammatory cytokine secretion. These effects sustain chronic inflammatory signals, which serve as a prerequisite for TLS initiation [42]. On the other hand, glucose metabolism also influences the function of T follicular helper cells (Tfh), which are critical for B cell activation and GC formation [43]. Adenosine triphosphate citrate lyase (ACLY) catalyzes the conversion of mitochondria-derived citric acid to acetyl coenzyme A and oxaloacetate. A recent study demonstrated that inhibition of ACLY leads to up-regulation of the chemokine CXCL13, thereby promoting B cell infiltration and facilitating the initiation of TLSs [44]. Therefore, enhancing the glycolytic activity of immune cells can indirectly influence the initiation and formation of TLSs.

In addition to directly acting on immune cells, glucose metabolism can also affect TLS initiation through other mechanisms [45]. First, elevated glycolysis leads to lactate accumulation, which not only alters the local pH but also acts on endothelial cells and fibroblasts to promote angiogenesis and matrix remodeling. Second, glucose metabolism can activate metabolic signaling pathways such as hypoxia-inducible factor 1-alpha, which in turn promotes the expression of chemokines and inflammatory cytokines, thereby strengthening lymphocyte recruitment and localization [46].

#### Lipid metabolism

Lipid metabolism is a fundamental biological process essential for maintaining cellular homeostasis, encompassing lipid uptake, synthesis, and oxidation [47]. Recent studies have demonstrated that lipid metabolism plays a critical role in regulating immune cell function and may be linked to the initiation of TLSs through these effects. Macrophages exhibit distinct polarization states depending on their lipid metabolic status: fatty acid oxidation (FAO) generally promotes differentiation toward the M2 phenotype, whereas dysregulated metabolism of saturated fatty acids and cholesterol can drive M1 polarization, thereby enhancing inflammation and antitumor activity [48]. The antigen-presenting capacity of DCs is likewise influenced by lipid metabolism, as excessive lipid droplet accumulation impairs their function and diminishes T cell stimulation [49]. For T cells, both activation and memory phases often rely on FAO to sustain energy supply.

TLS initiation depends on persistent inflammatory signaling, chemokine networks, and the coordinated recruitment of T and B cells [50,51]. Lipid metabolism indirectly facilitates this process by shaping the functional states of immune cells. Pro-inflammatory lipid mediators, such as oxidized LDL and arachidonic acid derivatives, can enhance the activity of DCs and macrophages, induce chemokine secretion, and thereby promote lymphocyte recruitment [52,53].

#### Amino acid metabolism

An increasing body of evidence indicates that amino acid metabolism plays a critical role in the initiation of TLSs [54]. Glutamine, as one of the most important nitrogen and carbon sources for immune cells, is markedly up-regulated in uptake and utilization upon T and B cell activation. Glutamine not only fuels the tricarboxylic acid (TCA) cycle but also activates the mTORC1 signaling pathway, which promotes lymphocyte proliferation and Tfh differentiation. Through these effects, glutamine contributes to the initiation and maturation of TLSs [55]. Tryptophan metabolism influences TLS function and composition indirectly through immunosuppressive pathways [56]. The indoleamine-2,3-dioxygenase-mediated kynurenine pathway markedly depletes local tryptophan levels, limiting excessive T cell activation, while kynurenine metabolites induce the differentiation of regulatory T cells (Tregs) [57]. This pathway prevents excessive inflammatory damage, during the early stages of TLS formation, by balancing the ratio of effector T cells to Tregs, thereby determining the immunological characteristics and persistence of TLSs. Arginine metabolism also plays a pivotal role in TLS structural remodeling and angiogenesis. Through the nitric oxide synthase pathway, arginine is converted into nitric oxide, which not only regulates inflammatory responses but also promotes angiogenesis and endothelial cell activation, providing essential conditions for TLS initiation [58].

#### Energy metabolism and redox

The primary function of mitochondrial metabolism is the conversion of energy, and its TCA and oxidative phosphorylation processes not only provide adenosine triphosphate (ATP) to the cell but also participate in signaling through metabolic intermediates [59]. Oxidative stress, as a product of metabolic abnormalities, has a bidirectional regulatory effect on TLS initiation. Reactive oxygen species (ROS) produced by NADPH oxidase can initiate TLS formation by activating NF-κB signaling and inducing the expression of ICAM1 and CXCL12. However, excessive ROS accumulation may inhibit FAO in LTo cells, thereby reducing the energy supply and affecting TLS maintenance. Acetyl-CoA acetyltransferase 1 (ACAT1) is a mitochondria-localized enzyme that primarily modulates protein function through acetylation and plays a key role in mitochondrial metabolism. In non-small cell lung cancer (NSCLC), ACAT1 acts as a metabolic regulator of TLSs and plays a pivotal role in the initiation phase. Mechanistic studies revealed that ACAT1 results in mitochondrial protein hypersuccinylation in lung tumor cells, thereby enhancing mitochondrial oxidative metabolism and ultimately inhibiting TLS formation.

In maintaining redox balance, glutathione metabolism plays a central regulatory role [60]. Glutathione is one of the most abundant intracellular antioxidants and sustains immune cell homeostasis by modulating the redox state. Recent studies have shown that glutathione is markedly accumulated in TLSs within the kidney [61]. Glutathione metabolism not only prevents excessive oxidative stress-induced damage to immune cells but also indirectly promotes immune cell activation by regulating signaling pathways, thereby creating favorable conditions for TLS initiation.

### Cell death

In recent years, cell death has also been recognized as one of the key factors in initiating TLS formation [62]. Cell death is essential for the renewal of normal tissues and the maintenance of systemic homeostasis. Different forms of cell death not only function in cell clearance and tissue repair but also shape the local immune microenvironment through mechanisms such as antigen release, exposure of damage-associated molecular patterns (DAMPs), and secretion of inflammatory mediators, thereby influencing TLS initiation. Studies have shown that in contexts such as tumors and chronic infections, the antigens and inflammatory signals generated by extensive cell death can act as critical driving forces for TLS initiation. For example, immunogenic cell death (ICD) has been demonstrated to promote TLS formation within the TMEs and enhance local antitumor immunity [62,63].

#### Necrosis

Necrosis is a typical form of non-programmed cell death characterized by plasma membrane rupture, leading to the direct release of intracellular contents into the extracellular environment and thereby triggering a strong inflammatory response [64]. Necrotic cells release large amounts of antigens and DAMPs, including calreticulin, high-mobility group box 1 (HMGB1), and ATP [65]. These molecules can be recognized and taken up by immune cells such as DCs and macrophages, thereby activating downstream inflammatory signaling pathways. At the same time, these DAMPs can induce the expression of various inflammation- and chemokine-related factors associated with TLS initiation, promoting the recruitment of lymphocytes to sites of inflammation [66]. For example, HMGB1 can bind to TLR4 on DCs, promoting DC maturation and the release of pro-inflammatory cytokines [67]. The molecular mechanisms triggered by necrosis are of great significance for TLS initiation. It is noteworthy that necrosis-associated inflammation exerts a dual effect: appropriate levels of antigen and inflammatory signals can promote TLS initiation and formation, favoring local immune responses, whereas excessive or uncontrolled necrosis may cause tissue damage and consequently alter the functional properties of TLSs.

#### Apoptosis

The process of apoptosis is accompanied by DNA degradation, chromatin condensation, cell membrane outgrowth, and apoptotic vesicle formation [68]. These changes ensure the orderly release of cellular contents and subsequent clearance by phagocytosis, thereby maintaining microenvironmental homeostasis. Apoptosis may be indirectly involved in TLS initiation through the release of signaling molecules that attract immune cell recruitment and promote antigen presentation and immune response initiation. It has been shown that apoptosis-related TMEs signatures are associated with TLS formation. Plasma cells contribute to the initiation phase of TLSs by activating CD74/CD44 or CD74/CXCR4 complexes within the MIF signaling pathway, thereby recruiting B cells, Tfh cells, and myeloid cells [69]. Notably, apoptosis in alveolar epithelial cells was abolished by blocking LTβR, a pivotal signaling component required for TLS initiation [70]. Furthermore, Dieudé et al. [71] demonstrated that apoptotic exosome-like vesicles up-regulate IL-17 secretion and promote TLS biogenesis.

#### Pyroptosis

Pyroptosis is a type of programmed cell death mediated by inflammatory responses, which occur mainly in the context of pathogenic infections, TMEs, and autoimmune diseases [72]. While direct evidence linking pyroptosis to TLS formation remains scarce, the potential association between them can be inferred through shared inflammatory pathways. Pyroptosis is mediated by Gasdermin proteins, which induce membrane pore formation and subsequent release of pro-inflammatory cytokines such as IL-1β and IL-18. These cytokines may promote immune cell infiltration to inflammatory sites, thereby providing inflammatory signals that could facilitate TLS initiation. For instance, pyroptotic cells release the HMGB1 protein, which acts extracellularly as a DAMP to promote CXCL12 production via TLR4 signaling [73]. Additionally, widespread pyroptosis occurs in periodontal inflammation, where IL-1β released by pyroptotic cells enhances the expression of chemokines including CCL2, CCL5, CXCL5, and CXCL12 [74].

#### Others

In addition to the aforementioned modes of cell death, other forms may also contribute to TLS initiation. Necroptosis, ferroptosis, and cuproptosis, as distinct types of cell death or stress responses, may participate in TLS initiation by regulating inflammation, antigen presentation, and immune cell recruitment [75].

## Cellular Interactions and Signaling in TLS Initiation

**Fig. 3.**
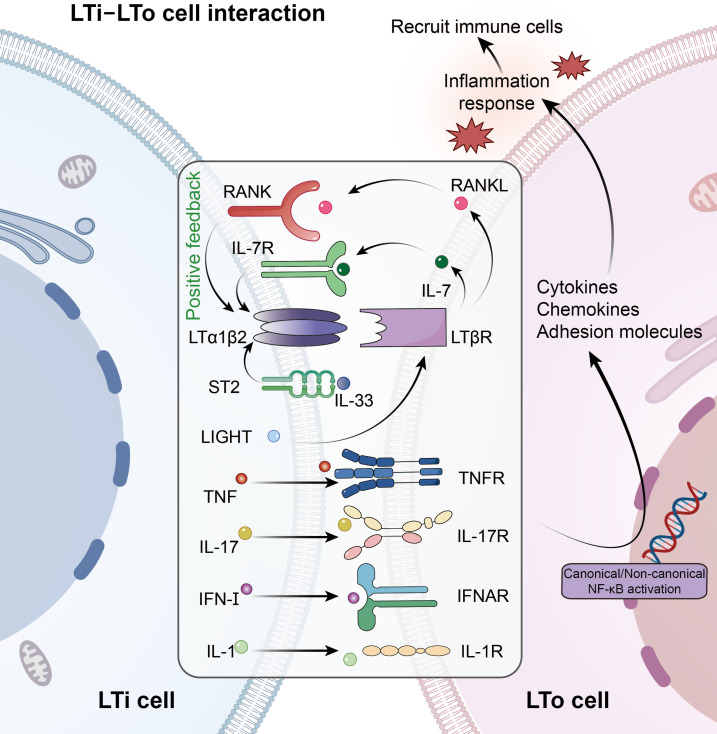
Cellular interactions and signaling in TLS initiation. In the inflammatory microenvironment, LTi cells interact with LTo cells through multiple signaling networks. These signals activate canonical and non-canonical NF-κB pathways in LTo cells, inducing cytokines, chemokines, and adhesion molecules to recruit immune cells and promote TLS initiation. IFN-I, type I interferon; IL, interleukin; LTi, lymphoid tissue inducer; LTo, lymphoid tissue organizer; LTα1β2, lymphotoxin α1β2; LTβR, lymphotoxin β receptor; RANK, receptor activator of nuclear factor κB; RANKL, receptor activator of nuclear factor κB ligand; TNF, tumor necrosis factor; TNFR, tumor necrosis factor receptor.

As previously mentioned, the initiation phase of TLSs not only relies on the canonical LTα1β2–LTβR pathway but also involves the synergistic activation of multiple signaling networks [76]. In this section, the focus is on the cellular interactions and signaling pathways that are involved in the initiation phase (Fig. 3). Causal and associative evidence for TLS initiation is distinguished and hierarchically summarized in Table 2.

**Table 2.** Evidence tiers for TLS initiation: causal interventions versus associative observations

Initiator	Model system	Representative readouts	Level of evidence	Reference
LTα1β2	Mouse	CCL19, CCL21, CXCL12, and CXCL13 induction; HEV formation.	Causal	[81,101]
LIGHT	Mouse	Chemokine induction; HEV formation.	Causal	[76,81]
TNF	Mouse; engineered stromal cultures	ICAM1/VCAM1 induction; immune cell recruitment.	Causal	[84]
IL-1	Mouse	NF-κB activation; immune cell recruitment.	Causal	[84]
IL-7	Mouse	IL-7/IL-7R signaling; amplification of LTα1β2 expression; CCL19/CCL21/CXCL13 induction.	Causal	[24,78]
IL-17	Mouse	Stromal activation; chemokine induction; immune cell recruitment.	Causal	[80]
IL-33	Mouse; human PDAC samples	IL-33-activated ILC2 expansion; LTα1β2 expression on ILC2; CCL19/CCL21/CXCL13 induction; HEV formation.	Causal (mouse); correlative (human)	[15]
IFN-I	Mouse; human tumor samples	Stromal activation; chemokine induction; HEV formation.	Causal; correlative	[83]
STING (cGAS–STING)	Mouse; human tumor datasets	Endothelial CCL5 induction; CCR5^+^CD8^+^ T cell recruitment; CXCL13 production; HEV formation.	Causal (mouse); correlative (human)	[85,86]

LTα1β2, lymphotoxin α1β2; TNF, tumor necrosis factor; HEV, high endothelial venule; IFN-I, type I interferon; PDAC, pancreatic ductal adenocarcinoma; ILC, innate lymphoid cell

### LTi–LTo core axis

LTi cells act as core initiators of TLS formation [77]. By secreting LTα1β2 and TNF, they bind to LTβR and TNFR on LTo cells, triggering cascading reactions. This process induces LTo cells to release chemokines and adhesion molecules, thereby establishing chemical gradients that recruit immune cells. Specifically, CXCL13 mobilizes B cells, whereas CCL19 and CCL21 recruit DCs and T cells toward inflammatory sites. Activation of the noncanonical NF-κB pathway proceeds relatively slowly. Notably, IL-7 and RANKL secreted by LTo cells act inversely on LTi cells to enhance LTi cell survival and LTα1β2 expression by binding to IL-7R and RANK [78]. This interaction forms a positive feedback loop of “LTi-LTo-LTi”, which ensures the continuous activation of the signaling pathway and the stabilization of the microenvironment.

### Synergistic effect of IL-17

In addition to the canonical LTα1β2–LTβR and TNF–TNFR pathways, IL-17 secreted by LTi cells (e.g., Th17 subsets) binds to IL-17R on LTo cell surfaces, thereby further amplifying the signaling cascade [79,80]. Upon IL-17 engagement, IL-17R activates both the canonical NF-κB pathway and mitogen-activated protein kinase signaling, thereby promoting the release of TLS-related chemokines such as CCL19 and CXCL13 [80]. Notably, IL-17 signaling also enhances the ability of LTo cells to respond to LTα1β2–LTβR signaling. Consequently, IL-17 signaling and LTα1β2–LTβR signaling synergistically form a positive feedback loop, thereby enhancing the stability of the TLS microenvironment.

### LIGHT–LTβR axis

LIGHT, a critical ligand for the LTβR, activates the canonical NF-κB signaling pathway by binding to LTβR on LTo cells [76]. This process drives transcriptional up-regulation of ICAM1 and VCAM1. Furthermore, the LIGHT–LTβR axis engages the noncanonical NF-κB pathway, inducing secretion of CXCL12 [81]. As a key chemokine for lymphocyte homing, CXCL12 binds to its receptor CXCR4, directing the migration of B cells, T cells, and other immune cells toward inflammatory sites.

### Alarmin-mediated IL-33/ST2 signaling

Beyond the classical LTi–LTo interactions, emerging evidence indicates that alarmin-mediated signaling contributes to TLS initiation [15]. IL-33, released from stressed or damaged stromal and endothelial cells, signals through its receptor ST2 expressed on LTi-like cells, including ILC2s. Engagement of the IL-33/ST2 axis enhances LTi cell activation and promotes LTα1β2 expression, thereby feeding into the LTβR-dependent stromal activation program.

### Early initiation of IFN and IL-1 signaling

IFN, a family of cytokines secreted by virus-infected cells or immune cells, exerts antiviral, antitumor, and immunoregulatory functions [82]. During the early phase of TLS initiation, IFN-I and IL-1 synergistically regulate the immune microenvironment, playing pivotal roles. IFN-I binds to the interferon alpha receptor on LTo cells, thereby activating the signal transducer and activator of transcription 1 pathway and inducing the secretion of chemokines such as CXCL9, CXCL10, CXCL13, CCL19, and CCL21 [83]. Meanwhile, IL-1 (e.g., IL-1β) enhances LTo cell inflammatory responses by activating the NF-κB pathway, further promoting CXCL13 secretion [84].

### Cross-regulation network

Emerging evidence indicates that TLS initiation does not rely on a single pathway but instead emerges from the integration of multiple partially overlapping regulatory axes. For instance, cross-talk between endothelial cells and T cells driven by the cGAS–STING pathway illustrates how innate immune sensing, chemokine signaling, and adaptive immune feedback cooperate to initiate TLS formation [85,86]. Together, these findings support a network-based model in which innate sensing, cytokine signaling, metabolic cues, and stromal activation are dynamically integrated to regulate the initiation of TLSs rather than operating as isolated linear pathways.

## Strategies for Regulating the Initiation of TLSs

**Fig. 4.**
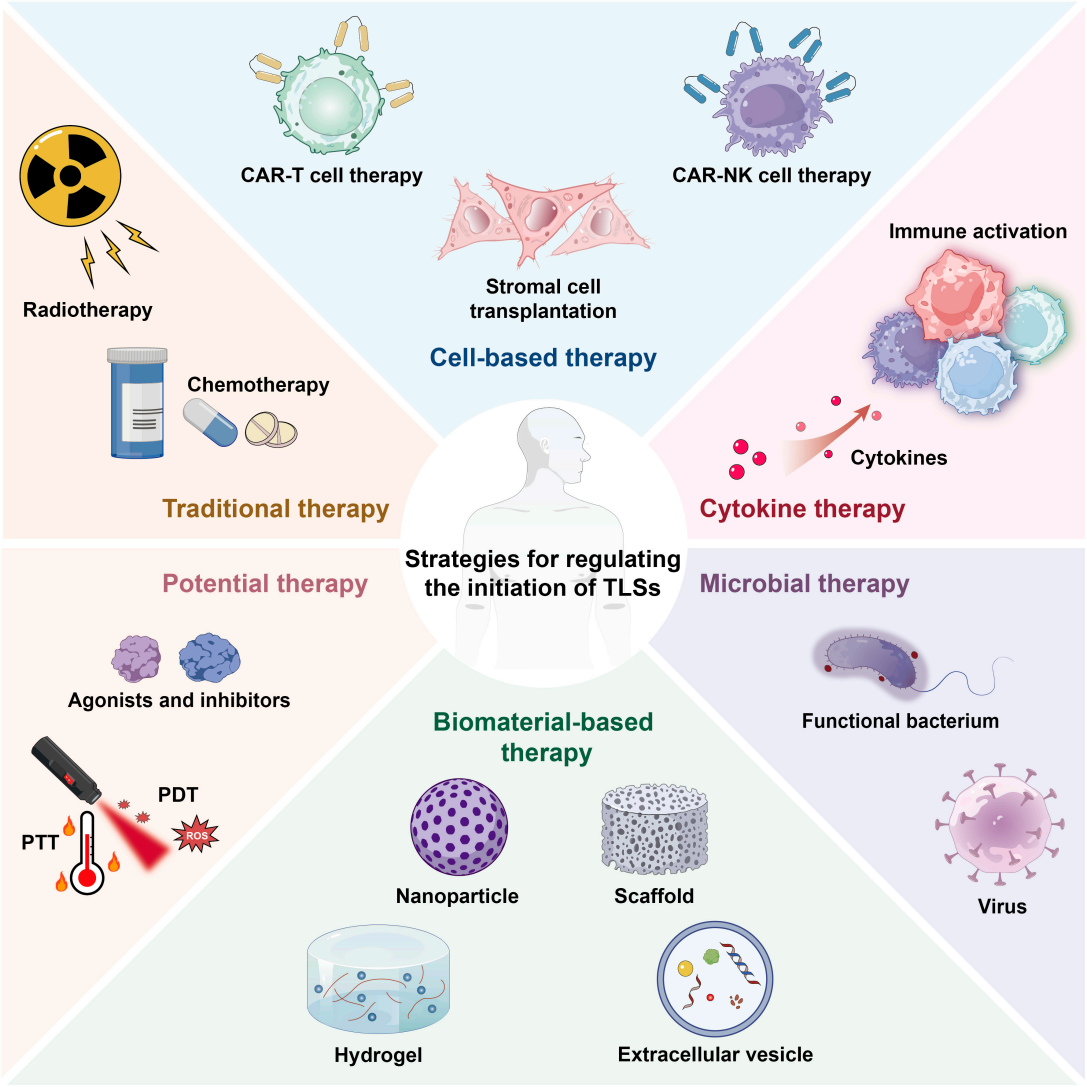
Regulatory strategies for TLS initiation. The regulatory strategies for TLS initiation include cytokine therapy, traditional therapy, cell-based therapy, microbial therapy, biomaterial-based therapy, and potential therapy, all of which regulate TLS initiation through distinct mechanisms. TLS, tertiary lymphoid structure; CAR, chimeric antigen receptor; NK, natural killer; PDT, photodynamic therapy; PTT, photothermal therapy; ROS, reactive oxygen species.

A growing body of evidence indicates that the presence of TLSs is closely associated with the immune microenvironment of various diseases, particularly playing important prognostic and therapeutic roles in cancer, infections, and autoimmune disorders [87]. How to induce TLS formation through interventional strategies has become a research hotspot in immunotherapy in recent years (Fig. 4). To provide a structured comparison of these regulatory approaches, Table 3 summarizes the efficacy, safety, and immune microenvironment compatibility of the strategies for regulating TLS initiation.

**Table 3.** Decision matrix comparing efficacy, safety, and immune microenvironment compatibility of TLS initiation strategies

Strategy	Efficacy	Safety	Immune microenvironment compatibility	Reference
Traditional therapy	Moderate (indirect via ICD and inflammation)	Dose-dependent immune suppression or toxicity; broad systemic effects.	Best in tumors where ICD improves antigen exposure.	[91]
Cell-based therapy	High (direct immune activation and cytokine release)	Immune overstimulation risk; limited persistence; complex manufacturing.	Immunologically “cold” or resistant tumors.	[94,95]
Cytokine therapy	High (direct chemokine-driven stromal activation)	Short half-life; off-target inflammation; systemic toxicity.	Tumors lacking chemokine niches.	[89]
Microbial therapy	Very high (strong innate activation, antigen exposure)	Risk of excessive inflammation or uncontrolled colonization.	Immune-excluded or antigen-poor tumors.	[98]
Biomaterial-based therapy	High (controlled release)	Dependent on biocompatibility and degradation characteristics.	Tumors lacking chemokine niches.	[100,101]
Potential therapy	Variable (context-dependent and often requires combination)	Immune overstimulation risk; variable tumor responsiveness.	Best used as combination enhancers.	[104–106]

ICD, immunogenic cell death

### Cytokine therapy

Cytokines play a central regulatory role in the development of TLSs [10]. Cytokine therapy, either through exogenous delivery or by enhancing specific cytokine levels in vivo, modulates immune cell recruitment, differentiation, and function. TLS initiation is closely associated with multiple cytokines: chemokines such as CXCL13, CCL19, and CCL21 are essential for lymphocyte migration, while lymphotoxin signaling activates stromal cells through LTβR to induce the expression of adhesion molecules and secondary signaling mediators, thereby facilitating the formation of stable follicle-like structures [88]. In a mouse melanoma model, injection of CXCL13 and CCL21 led to a marked increase in TLS formation [89]. However, many cytokines exhibit limited stability in vivo, requiring repeated administration or supraphysiological dosing, which increases toxicity risk and variability in treatment efficacy. To overcome these limitations, strategies such as localized delivery systems and engineered cytokine variants with prolonged half-life are being explored to enhance safety and improve delivery precision.

### Traditional therapy

Radiotherapy and chemotherapy have traditionally been regarded as approaches that directly kill tumor cells, but recent studies have revealed their important roles in modulating the immune microenvironment [90]. Radiotherapy induces DNA damage and oxidative stress through ionizing radiation, leading to ICD. During this process, cells release DAMPs such as ATP and HMGB1, which activate DCs and enhance antigen presentation, thereby gradually promoting the formation of TLS-like structures [90]. Similarly, chemotherapeutic agents such as doxorubicin and oxaliplatin can also induce ICD, thereby enhancing antigen exposure and immune responses [91]. Consistent with this notion, in hepatocellular carcinoma, hepatic arterial infusion chemotherapy with oxaliplatin, leucovorin, and fluorouracil has been shown to considerably enhance TLS formation within the TMEs [92]. It is important to emphasize that the effects of radiotherapy and chemotherapy on TLS initiation are often dose- and time-dependent: low-dose fractionated radiotherapy is considered more favorable for immune activation, whereas single high-dose irradiation may lead to immune suppression [93]. However, excessive cytotoxic exposure may also damage TLS-supportive stromal and immune cells, limiting TLS induction and long-term immune benefit; therefore, emerging strategies such as dose optimization, fractionated schedules, and combination with immunotherapy are being explored to balance cytotoxic effects and immune activation. Thus, traditional therapies not only are cytotoxic modalities but also can indirectly initiate TLS formation by regulating cell death, providing new opportunities for immunotherapeutic strategies.

### Cell-based therapy

Cell-based therapies are designed to target this mechanism by modulating the immune microenvironment through exogenous cell transplantation or engineered cell modification, thereby initiating TLS formation [94]. Among these strategies, stromal cell transplantation involves the introduction of exogenous fibroblast-like stromal cells into target tissues, where they directly provide the chemokines and adhesion molecules necessary for TLS initiation [94]. Chimeric antigen receptor (CAR)-T cell therapy, which modifies T cells with CARs, eliminates target cells with high specificity while simultaneously releasing large amounts of cytokines, thereby promoting the activation of stromal and endothelial cells and indirectly inducing TLS formation [95]. Similarly, CAR-NK cell therapy enhances the targeting ability of NK cells through genetic modification, enabling them to kill target cells while further promoting antigen presentation and lymphocyte recruitment [96]. Despite these advantages, these approaches remain limited by manufacturing complexity, persistence issues, and immune toxicity. Emerging controlled activation and localized delivery strategies aim to improve safety and feasibility.

### Microbial therapy

Microorganisms, owing to their intrinsic immunogenicity, are regarded as potent initiators of TLSs [31]. OVs selectively lyse target cells while simultaneously releasing large amounts of antigens, and their nucleic acids and proteins can be recognized by TLRs, thereby inducing type I interferons and various chemokines (CXCL13, CCL19, and CCL21) to initiate TLS formation [97]. However, OVs may cause off-target toxicity to normal tissues; optimizing viral tropism through genetic engineering can reduce this. Similarly, engineered bacteria can also be exploited to trigger TLSs. Bacteria naturally possess strong immunogenicity, with cell wall components and flagellin proteins serving as PAMPs that continuously stimulate the innate immune system, inducing inflammatory responses and chemokine expression [98]. Yet, engineered bacteria might trigger excessive immune reactions or be cleared rapidly by the host. Using biocompatible coatings or adjusting bacterial dosage can help. For example, Su et al*.* designed an engineered bacterium to initiate TLS formation. They functionally modified *Escherichia coli* by encapsulating cytokines within the bacteria, which, through its inherent tumor-targeting capacity, colonized the TMEs and elicited potent antitumor effects [99]. Overall, microbial therapies provide both antigens and danger signals, thereby creating an inflammatory microenvironment conducive to TLS formation.

### Biomaterial-based therapy

Biomaterials, through their controllable release properties and capacity to provide structural support, can mimic the microenvironment required for natural lymphoid organogenesis and thus exhibit unique advantages in TLS initiation [100]. Nanoparticles can serve as drug carriers to deliver cytokines, antigens, or agonists to specific tissues. Hydrogels, owing to their excellent biocompatibility and 3-dimensional network structure, can act as reservoirs and sustained-release systems for immune molecules, while simultaneously providing a physical scaffold for cell migration and lymphocyte recruitment [101]. Extracellular vesicles, as natural biological nanocarriers, contain surface ligands and signaling molecules that can be engineered to deliver immune-related factors, thereby exerting therapeutic effects in target tissues [102]. Studies have shown that radiotherapy-derived stem cell exosomes can activate the cGAS–STING pathway and release large amounts of cytokines, ultimately triggering TLS formation [103]. Nonetheless, several issues still need to be addressed, including long-term biocompatibility, degradation behavior, and the heterogeneity of host responses. Furthermore, with the continuous advancement of biomaterials, intelligent materials are anticipated to achieve on-demand drug release in inflammatory or pathological environments, offering new avenues for the precise induction of TLSs.

### Potential therapy

In addition to the aforementioned strategies, several potential therapeutic approaches have also been proposed to contribute to TLS initiation [96]. Photodynamic therapy (PDT) and photothermal therapy (PTT) induce ICD in tumor cells through the generation of local ROS or hyperthermia, thereby activating DCs and up-regulating related cytokines, which create favorable conditions for TLS initiation [104]. However, PDT/PTT may cause off-target tissue damage; optimizing light parameters and using targeted delivery can mitigate this.

Moreover, small-molecule agonists or inhibitors have recently been suggested as potential tools for TLS initiation [105]. For instance, TLR agonists can stimulate antigen presentation and amplify local inflammatory responses [105]. LTβR signaling is a key regulator of lymphoid organogenesis, and LTβR agonists can promote TLS formation by up-regulating chemokines and activating immune cells, yet the long-term effects of LTβR agonists are unclear [106]. Notably, emerging evidence indicates that STING agonists or ICB may induce TLS formation concomitantly with the expansion of immunosuppressive B cell subsets [107]. These potential strategies, particularly when combined with cytokine-based or biomaterial-based therapies, hold promise for further improving the efficiency and stability of TLS initiation.

**Fig. 5.**
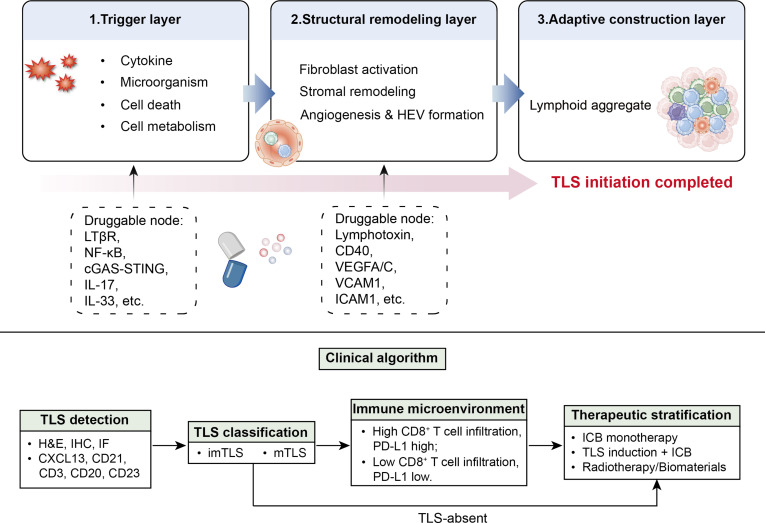
Three-tier mechanism and clinical algorithm of TLS initiation. An integrated schematic summarizing the 3-tier map of TLS initiation, including upstream initiating cues, cellular and stromal regulatory networks, and therapeutic intervention strategies. The lower panel outlines a clinical algorithm for therapeutic stratification to guide patient stratification and immunotherapeutic decision-making. TLS, tertiary lymphoid structure; mTLS, mature tertiary lymphoid structure; imTLS, immature tertiary lymphoid structure; HEV, high endothelial venule; VEGF, vascular endothelial growth factor; ICB, immune checkpoint blockade; IF, immunofluorescence; IHC, immunohistochemistry; H&E, hematoxylin and eosin; LTβR, lymphotoxin β receptor.

These emerging interventional strategies highlight the need to view TLS initiation within a broader mechanistic and translational framework. To integrate the mechanistic basis of TLS initiation with its therapeutic regulation and clinical relevance, a unified schematic was generated to summarize the 3-tier initiation framework, druggable regulatory nodes, and a clinical algorithm for TLS evaluation and therapeutic stratification (Fig. 5).

## Conclusions and Prospects

**Fig. 6.**
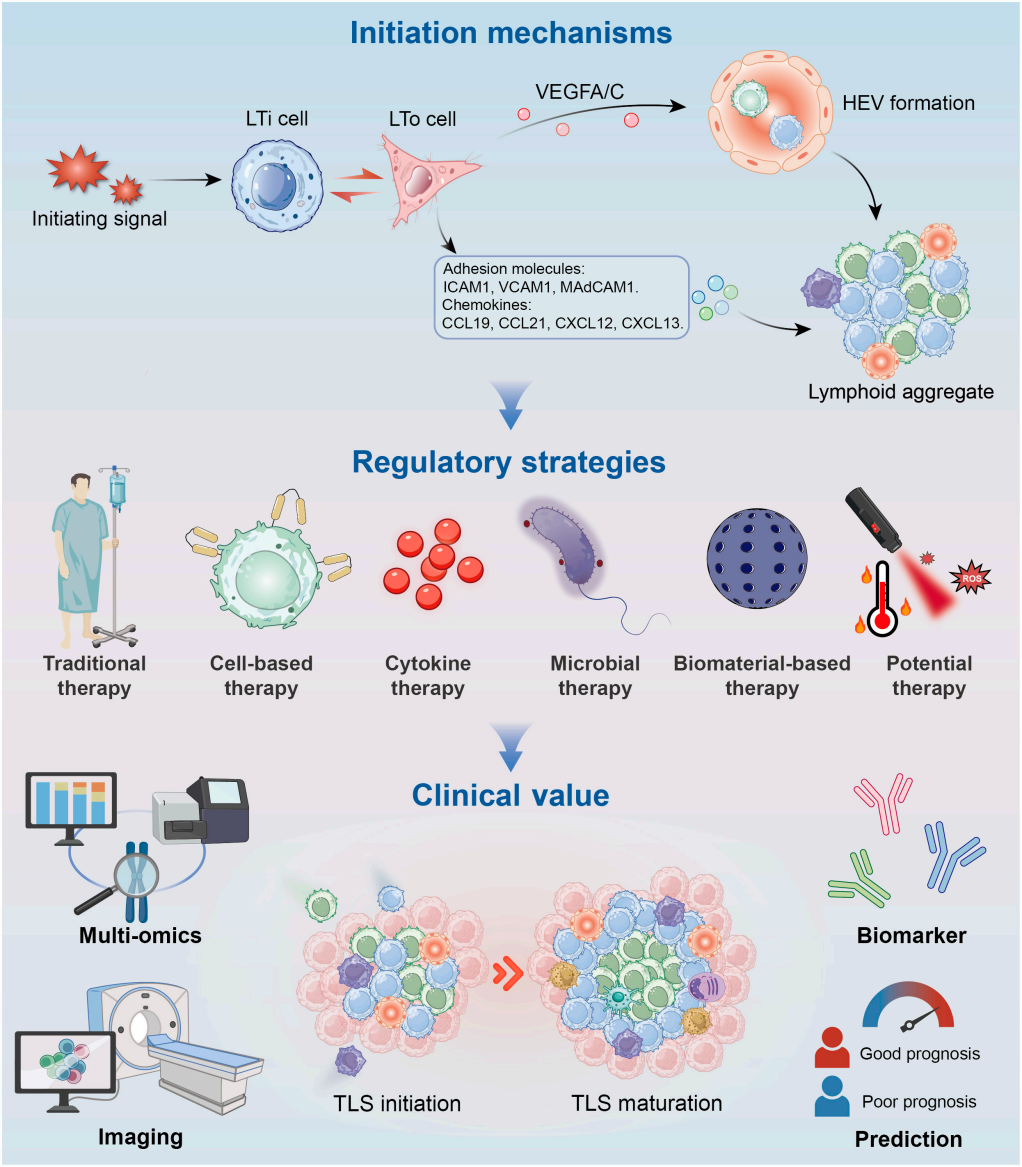
Mechanisms, regulation, and clinical value of TLS initiation. Graphical summary depicting the key molecular and cellular mechanisms driving TLS initiation, the regulatory strategies targeting this process, and the associated clinical implications in tumor immunity and immunotherapy. TLS, tertiary lymphoid structure; LTi, lymphoid tissue inducer; LTo, lymphoid tissue organizer; VEGF, vascular endothelial growth factor; HEV, high endothelial venule.

The initiation of TLSs is a tightly regulated and highly organized process involving the coordinated actions of multiple cell types [80]. In Fig. 6, the initiation mechanisms, regulatory strategies, and clinical value of TLSs are summarized. Although key mechanisms such as LTβR signaling, HEV ontogeny, and chemokine secretion are increasingly understood, the upstream triggers remain incompletely defined. Future work integrating longitudinal spatial transcriptomics, single-cell multi-omics, and computational modeling will be essential to map the temporal architecture of TLS initiation and identify targetable regulatory nodes.

TLS-associated biomarkers are emerging not only as indicators of immune activation but also as early pharmacodynamic readouts [108]. Clinically, TLS detection is primarily performed using hematoxylin and eosin (H&E) staining and multiplex immunohistochemistry/immunofluorescence (IHC/IF) to assess TLS density, location, and mTLS status, while transcriptomic TLSscore and spatial transcriptomics provide complementary molecular and spatial stratification [109–111]. Prognostic and predictive cutoffs are typically defined by TLS density, mTLS positivity, or TLSscore-high versus TLSscore-low classification. In molecular imaging, computed tomography (CT), multiparametric magnetic resonance imaging (MRI), and positron emission tomography (PET) can achieve real-time or high-resolution visualization of TLSs [112,113]. The corresponding detection methods and their predictive stratification criteria are systematically summarized in Table 4.

**Table 4.** Multilayered frameworks for TLS detection and heterogeneity assessment, and translational clinical evaluation across platforms

Detection level	Representative methods	Key readouts	Maturity classification	Quantitative measures	Spatial information	Reproducibility	Clinical applicability	Reference
Conventional histology	H&E; IHC; IF	TLS presence; B/T cell zones; FDC network; HEVs (PNAd, CD31); GC markers (BCL6, AID); chemokines (CCL19/21, CXCL12/13), etc.	Yes	TLS number; area; density	Local	High	Widely used	[7]
Molecular metrics	TLS score	12-chemokine gene; TLS imprint; inflammatory and stromal signaling proteins.	Yes/No	Expression level; enrichment index	None	High	Prognostic and predictive biomarker	[19]
Spatial transcriptomics	Visium; Xenium; MERFISH; HDST; GeoMX DSP; Stereo-seq	Chemokines; HEV co-localization; spatial organization of immune-stromal niches.	Yes	Spatial TLS density	High	Moderate	Emerging translational value	[18,56,109]
Spatial proteomics	Phenocycler-fusion; IMC; MIBI	Spatially resolved protein expression and abundance; immune-stromal co-localization.	Yes	Protein abundance; cell density	High	Moderate	Emerging translational value	[110,111]
Multimodal imaging	CT/PET/MRI with TLS-related tracers	Whole-body TLS distribution.	Functional inference	Volumetric uptake	3D	Moderate	Non-invasive longitudinal monitoring	[112,113]

TLS, tertiary lymphoid structure; LTi, lymphoid tissue inducer; LTo, lymphoid tissue organizer; VEGF, vascular endothelial growth factor; HEV, high endothelial venule; IF, immunofluorescence; IHC, immunohistochemistry; H&E, hematoxylin and eosin; GC, germinal center; CT, computed tomography; MRI,magnetic resonance imaging; PET, positron emission tomography

The initiation of TLSs represents a therapeutically actionable checkpoint in tumor immunity rather than a passive biological phenomenon [114]. Metrics such as immature TLS (imTLS)-to-mature TLS (mTLS) conversion, HEV expansion, and chemokine-rich stromal signatures may serve as response-adaptive endpoints in interventional trials. The mTLS status has been consistently associated with favorable prognosis and enhanced responsiveness to immunotherapy across multiple cancer types [115]. In colorectal cancer, the presence of intratumoral mTLS correlates with prolonged overall survival and improved response to PD-1/PD-L1 blockade [116]. Similar survival benefits have been reported in melanoma, NSCLC, breast cancer, muscle-invasive bladder cancer, prostate cancer, renal cell carcinoma, and liver cancer, in which mTLS density is associated with sustained antitumor immune activation and durable clinical responses [87,115,117–121]. Prototype clinical trial models are now conceptually feasible. A neoadjuvant strategy that combines TLS induction and ICB may precondition immune-cold tumors prior to checkpoint blockade, thereby improving response rates. Similarly, a metastatic “immune re-warming” model could enable TLS-directed agents to convert non-responsive metastatic lesions into immunologically permissive sites.

Taken together, accumulating evidence supports a conceptual framework in which TLSs evolve through a continuous trajectory from initiation to maturation and ultimately to immunotherapy responsiveness. Among these stages, the initiation phase represents a decisive and therapeutically actionable checkpoint, as it establishes the chemokine niches, stromal activation programs, and vascular gateways that determine whether effective immune architecture can be assembled within tumors. Successful initiation enables subsequent TLS maturation, together establishing the immunological foundation that underpins favorable responses to ICB.
